# Endoluminal Vacuum Therapy as Effective Treatment for Patients with Postoperative Leakage After Metabolic Bariatric Surgery—A Single-Center Experience

**DOI:** 10.1007/s11695-024-07367-2

**Published:** 2024-07-24

**Authors:** L. Gensthaler, M. Stauffer, J. Jedamzik, C. Bichler, L. Nixdorf, P. Richwien, J. Eichelter, F. B. Langer, G. Prager, D. M. Felsenreich

**Affiliations:** Division of Visceral Surgery, Department of General Surgery, Medical University of Vienna, Währinger Gürtel 18-20, 1090 Vienna, Austria

**Keywords:** Bariatric surgery, RYGB, Revisional surgery, Endoluminal vacuum therapy

## Abstract

**Background:**

Metabolic bariatric surgery (MBS) is standardized and safe. Nevertheless, complications such as anastomotic leakage (AL) or staple-line leakage (SLL) can occur. In upper GI or colorectal surgery, endoluminal vacuum therapy (EVT) offers a therapeutic alternative to revisional surgery. Data on EVT in patients with leakage after MBS remain scarce. The aim of this study is to evaluate the efficacy of EVT and its potential as endoscopic alternative to revisional surgery.

**Material and Methods:**

All patients treated for AL or SLL with EVT after MBS between 01/2016 and 08/2023 at the Department for General Surgery, Medical University Vienna, were included in this retrospective, single-center study. Therapeutic value of EVT as management option for acute postoperative leakage after MBS in daily practice was evaluated. Statistical analyses were performed descriptively.

**Results:**

Twenty-one patients were treated with EVT within the observational period of 7 years. In 11 cases (52.4%), the index surgery was a primary bariatric intervention; in 10 cases (47.6%), a secondary surgery after initial MBS was performed. Favored approach was a combination of revisional surgery and EVT (*n* = 18; 85.7%), intermediate self-expanding metal stent (SEMS) in 16 (76.2%) cases. EVT was changed six times (0–33) every 3–4 days. Mean EVT time was 25.1 days (3–97). No severe associated complications were detected and EVT showed an efficacy of 95.2%.

**Conclusion:**

This small case series supports the trend to establish EVT in daily clinical practice when revisional surgery after MBS is needed, thus preventing further reoperation and reducing associated morbidity and mortality in critically ill patients.

**Graphical Abstract:**

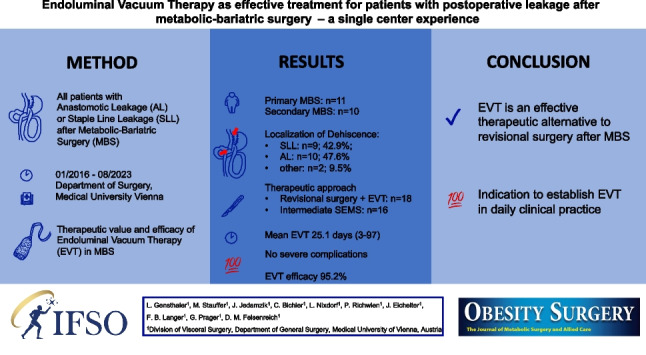

## Introduction

Metabolic/bariatric surgery (MBS) is the most successful therapeutic option for treating patients with obesity [1, 2]. Due to increasing incidence of obesity and therefore, increasing necessity for MBS worldwide, MBS has become highly standardized and safe [3]. Nevertheless, knowledge about the management of MBS-associated complications is of major importance since patients with obesity are especially at risk for perioperative complications due to their associated comorbidities [4–7]. Even though anastomotic leakage (AL) and staple-line leakage (SLL) after MBS are rare (reports vary between 1.5 and 5.6%), those complications affect patients’ perioperative outcome immensely [8–10]. Especially in the early postoperative phase, clinical symptoms like tachycardia, hypotonia, fever, or severe pain are alarming indicators. At the MUV, not only a wide spectrum of primary MBS procedures, but also revisional surgeries for critically ill patients experiencing complications after MBS (often referred from other bariatric centers) are performed. Since revisional surgeries are very challenging and demand high surgical expertise, additional tools for the treatment of complications, such as endoscopic vacuum therapy (EVT) or self-expanding metal stent (SEMS) therapy, have been established [11, 12]. Additionally, or even instead of an early surgical approach, EVT, as described in studies on upper GI leakage or pouch leakage in colorectal surgery, represents a very promising endoscopic and minimally invasive therapeutic alternative [13–16]. To date, only a few case series of patients treated with EVT after complications in MBS have been reported, but with promising results [17]. The aim of this study was to evaluate the clinical relevance and efficacy of EVT for patients with postoperative complications after MBS at a specialized, high-volume MBS center.

## Patients and Methods

All patients treated with EVT due to AL or SLL after MBS at the MUV between 01/2016 and 08/2023 were included in this case series. This analysis was approved by the local ethics review committee (Ethics committee, Medical University of Vienna, Nr. 1279/2022). Patients’ data were collected retrospectively and pseudonymized. Data used for this study were routinely assessed during patients’ medical treatment. Patients who underwent index surgery at the MUV, as well as those transferred from other bariatric centers specifically for the treatment of leaks following MBS, were included in this case series. Revisional surgeries and endoscopic interventions were always performed by the same group of surgeons, specialized in MBS.

### Patient Characteristics

Basic patient characteristics such as gender, age, BMI, and comorbidities were collected. Index surgery was either a primary MBS procedure or a revisional MBS due to various associated complications such as weight regain, reflux, or anastomotic ulcer. The surgical technique was subdivided in sleeve gastrectomy (SG) and gastric bypass procedures (RYGB, Roux-en-Y gastric bypass; SADI-S, single-anastomosis-duodeno-ileale bypass with sleeve gastrectomy) as well as revisional surgery. The location of primary surgery was defined as MUV or an outward bariatric center. Admission to ICU (intensive care unit) and duration of ICU admission, as well as the duration of hospital admission at the MUV, were evaluated. Persistent symptoms and necessity for reinterventions were evaluated during patients’ follow-up visits. Mortality was defined as patients’ death within 90 days since the index surgery and afterwards.

### Clinical Characteristics

Perioperative data such as the onset of symptoms, defined as the period between surgery and the development of symptoms suggesting leakage, or the interval between index surgery and diagnosis of leakage were evaluated.

### Definition and Diagnosis of Leakage

Occurrence of leakage was categorized according to the classification by Rosenthal et al., as acute leakage, diagnosed within 0–7 days, early leakage between weeks 1 and 6, late leakage identified between weeks 7 and 12, and as chronic leakage, occurring beyond week 12 [18]. Since diagnosis was not only based on clinical parameters but also based on a positive contrast-swallow X-ray or contrast-swallow CT -(computed tomography) scan, efficacy was evaluated. If drains were inserted, leakage recurrence was assessed the following days during an EVT pause with a methylene-blue swallow test, while observing the drainage at the anastomosis site. Clinical symptoms such as tachycardia > 100 bpm (beats per minute), hypotension, fever, and an extended need for pain medication, as well as elevated inflammation parameters, were also taken into consideration for indicating revisional surgery, especially in the early postoperative phase. Endoscopy and its efficacy for diagnosing leakage were evaluated as well. The site of the dehiscence as well as associated complications is reported.

### Therapeutic Procedure and Outcome

In terms of revisional surgery, the therapeutic approach was chosen individually, ranging from standalone EVT to a combination of EVT and diagnostic laparoscopy to the necessity of SEMS therapy. When a leak was identified during revisional surgery, an air-fluid leak test was performed to further detect the site of the leakage or assess the integrity of the staple line or anastomosis. This involved insufflating CO_2_ by upper GI endoscopy while flushing the observed area with NaCl (sodium chloride). Subsequently, the leak was overstitched, and easy-flow drains were positioned precisely. Furthermore, exact placement of EVT was carried out using an endoscopic overtube or the pull-through technique. If EVT is placed using the overtube technique, the sponge is placed intraluminal right next to the dehiscence. In terms of using the pull-through technique, presence of precisely placed easy-flow drains is obligatory. While placing a guided wire through the drainage canal and connecting it to the endoscopically placed sponge, precise placement into the leakage/cavity is possible.

EVTs were replaced every 3–4 days and constant vacuum or slurp mode of − 60 to – 80 mmHg was used in all patients. Duration of EVT (median, days) was assessed. After consultation by a nutritionist, all patients received parenteral nutrition adapted to their caloric demand during EVT. As soon as SEMS was applied, moderate enteral nutrition was added. EVT-associated complications, such as dislocation, insufficient drainage, bleeding, intolerance of EVT, or patient’s non-compliance, were evaluated. Proper EVT placement and closure of the leak were defined as therapeutic success. In each patient, therapeutic strategy was discussed regularly within the team and adapted individually. Therefore, parameters leading to a change in therapeutic strategy were as heterogenous as the patient’s condition and clinical presentation. The following paragraph includes a few considerations for adapting the therapeutic approach in individual patients.

EVT was replaced by SEMS if there was a high output and huge cavity with no sufficient drainage. If there was a cavity with easy-flow drains placed in a previous revision, pull-through EVT placement was preferred. The treatment of a blowout fistula after SG or SADI-S was to place SEMS first to achieve adequate transpyloric outlet, then EVT for wound granulation. In case of small chronic fistula remaining after sufficient EVT, additional SEMS therapy was applied in some cases individually. Furthermore, patients’ compliance regarding SEMS tolerance and compliance for EVT were always taken into consideration*.*

### Statistical Analysis

Patient population was characterized by conducting descriptive statistics for preoperative and postoperative parameters. Categorical variables were presented as numbers (*n*) and proportions (%), continuous variables as median with range or mean with standard deviation. SPSS version 25 for Mac (IBM, Armonk, NY, USA) was used for statistical calculations.

## Results

### Patient Characteristics

Within an observational period of 7 years (01/2016−08/2023), 21 patients after primary/revisional MBS were treated with EVT due to postoperative complications. Eighteen (18/21; 85%) of the observed patients were female and the mean age was 41.9 (19–69) years. Mean BMI at the time of first EVT placement was 36 kg/m^2^ (median: 34,6 kg/m^2^, IQR: 21–60 kg/m^2^), and patients had various obesity-associated comorbidities such as diabetes mellitus (DM II, *n* = 6/21; 28%) and arterial hypertension (*n* = 7/21; 33.3%). Nicotine abuse was reported by nine patients (*n* = 9/21; 42.9%). In ten cases (*n* = 10/21; 47.6%), the index surgery was a primary bariatric intervention; in eleven cases (*n* = 11/21; 52.4%), a secondary surgery after initial MBS was performed. Eleven patients (*n* = 11/21; 52.4%) initially underwent surgery at the MUV. In ten cases (*n* = 10/21; 47.6%), the index surgery was performed at outward bariatric centers and patients were transferred to the MUV for further treatment due to postoperative complications. In six of those cases, first revisional surgery and additional SEMS placement were performed at outward bariatric centers, before the patients were transferred to the MUV due to persistent leakage. At the time of admission, two patients suffered from severe liver dysfunction, represented by elevated cholestatic parameters, altered clotting time, and ascites. Two patients suffered from hypoabsorption and malnutrition (vitamin E and electrolyte deficiency and hypoalbuminemia) due to short bowel syndrome at the time of admission at the MUV. Further information is demonstrated in Table 1.

**Table 1:** Patient characteristics

	*n* (% or SD)
Sex	
Male	3 (14.3%)
Female	18 (85.7%)
Age (years)	41.9 (±15.4)
BMI (kg/m^2^)	36 (±9.5)
Comorbidities at the time of admission	
Type 2 diabetes	6 (28.6%)
Arterial hypertension	7 (33.3%)
Nicotine abuse	9 (42.9%)
Hyperlipidemia	5 (23.8%)
Sleep apnea	4 (19.0%)
Other*	12 (57.1%)
Index surgery	
Primary	9 (42.8%)
Secondary	12 (57.2%)
Surgical technique	
Sleeve gastrectomy	4 (19.0%)
Gastric bypass	6 (28.6%)
RYGB	5 (23.8%)
SADI-S	1 (4.7%)
Revisional surgery	11 (52.4%)
Location of primary surgery	
Medical University of Vienna	11 (52.4%)
Outward bariatric center	10 (47.6%)

*IQR*, interquartile range; SD, standard deviation; *BMI*, body mass index; *MUV*, Medical University of Vienna; *RYGB*, Roux-en-Y gastric bypass; *SADI-S*, single-anastomosis-duodeno-ileale bypass with sleeve gastrectomy; *other: steatosis hepatitis, hypothyroidism, depression, gastroesophageal reflux disease, bronchial asthma

### Clinical Characteristics

Mean time from index surgery to diagnosis of the leak was 9 days (0–38). Acute leakage (days 0–7) occurred in 15 cases (*n* = 15/21; 71.5%), early leakage (weeks 1–6) in six cases (*n* = 6/21; 28.5%). No late or chronic leak was detected. Contrast-swallow CT-scan or swallow-X-ray was a sufficient diagnostic tool in 18 cases, whereas in three cases, no radiologic imaging, but immediate reintervention was performed. All patients had endoscopy during their first revision, which proved 100% success (21/21) at detecting leaks. The dehiscence was localized at the most upper part of the staple line or the esophago-gastric junction (EGJ) in ten cases (*n* = 10/21; 47.6%; 4 SG, 1 SADI-S, 2 YRGB, 3 pouch revisions) and in nine cases (*n* = 9/21; 42.8%) at the gastro-jejunostomy after primary or revisional MBS. In two complex cases, leakage was detected at the esophago-jejunostomy and esophago-colostomy (colonic interposition) and treated with EVT respectively. In five patients (*n* = 5/21; 23.8%), accompanied complications, such as pleural empyema/fistula and mediastinitis, occurred within the treatment period. For additional information, refer to Table 2.

**Table 2:** Clinical characteristics

	*n* (% or SD)
Interval between MBS + symptoms (days)	4.5 (0–16)
Interval between MBS + diagnosis of leak (days)	9.0 (0–38)
Diagnosis of leak during index stay° (yes)	19 (90.5%)
Time of diagnosis^§^	
Acute (days 0–7)	15 (71.5%)
Early (weeks 1–6)	6 (28.5%)
Late (weeks 7–12)	0 (0%)
Chronic (> week 12)	0 (0%)
Detection of leak in CT scan	18 (85.7%)
Endoscopy at 1^st^ revision	
Performed	21 (100%)
Detection of leak in endoscopy (yes)	21 (100%)
Localisation of dehiscence	
EGJ staple line*	10 (47.6%)
Gastro-enterostomy	9 (42.8%)
Esophago-jejunostomy	1 (4.8%)
Esophago-colostomy (Colonic interposition)	1 (4.8%)
Accompanied complications during leak/treatment	
Liver dysfunction	2 (9.5%)
Hypoabsorption	2 (9.5%)
Gastro-pleural fistula/empyema	5 (23.8%)

*IQR*, interquartile range; SD, standard deviation; *MBS*, metabolic bariatric surgery; *CT*, computed tomography; *EGJ*, esophago-gastric junction; range days: median (minimum–maximum)

*Most upper part of the staple line in patients after sleeve gastrectomy, SADI-S, pouch revision

°Leakage was diagnosed while patients were still in hospital care

^**§**^According to the classification by Rosenthal et al. [18]

### Therapeutic Procedure and Outcome

Seven (0–38) days in median passed between index surgery and first surgical revision and median 27.5 (0–89) days until EVT was initially applied. Individualized revisional surgery, such as diagnostic laparoscopy, endoscopy, suturing of the leakage, precise drainage, and EVT, was necessary for most cases (*n* = 18/21; 85.7%). In three cases (*n* = 3/21; 14.3%), standalone EVT was the therapeutic approach of choice. SEMS was applied in 16 (*n* = 16/21; 76.2%) cases pre- or post-EVT as a means to change or optimize therapeutic strategy individually. Of those, SEMS was placed at outward MBS centers six times before patients were transferred to the MUV and treated with EVT. In five cases, SEMS was applied prior to EVT and placed transpyloric when a blowout leakage/fistula in patients after SG was identified to create a low-pressure system and achieve adequate outflow. In five cases, SEMS was used after sufficient EVT, when wound granulation was achieved to prevent the development of a stenosis. If EVT was applied, it was replaced in median six times (0–33) with changes of the sponge every 3–4 days. EVT was placed endoscopically in 16 cases (*n* = 16/21; 76.2%) and via pull-through technique in five cases (*n* = 5/21; 23.8%). The mean duration of EVT was 25.7 days (± 22.4). No severe complications associated with EVT were detected; nevertheless, EVT dislocation was observed in one case and EVT had to be interrupted due to lack of patients’ compliance in three cases.

In 18 cases (*n* = 18/21; 85.7%), specialized treatment at the ICU was necessary with a mean length of stay of 26.4 days (± 25.2). The mean length of the hospital stay was 77.3 days (± 61.2). Additionally, all patients received intravenous parenteral nutrition and antimicrobial therapy during their treatment.

Median follow-up was 780.3 days (99–2176 days). During that follow-up period, three patients (3/21; 14.3%) developed dysphagia, resulting in endoscopic pneumatic dilatation at the location of previous dehiscence. Eight patients (*n* = 18/21; 38.0%) were diagnosed with depression with the necessity for medical treatment. Four patients (*n* = 4/21; 19%) died during the follow-up period, and three of them passed away 339 (238–944) days after successful treatment of the leak. One patient passed away due to postoperatively developed short bowel syndrome, potassium deficiency, and cardiac arrest; one patient died due to bacterial uremia and following sepsis; and one patient died due to kidney dysfunction but refusal of dialysis. One patient died after unsuccessful EVT in-hospital due to persistent esophago-cutaneous fistula and following necrotizing fasciitis after a complex course of revisional surgeries with the necessity of performing an colonic interposition. Thus, sufficient granulation tissue and successful outcome (complete closure of the leak) were observed in 20 cases (*n* = 20/21; 95.2%).

**Fig. 1:**
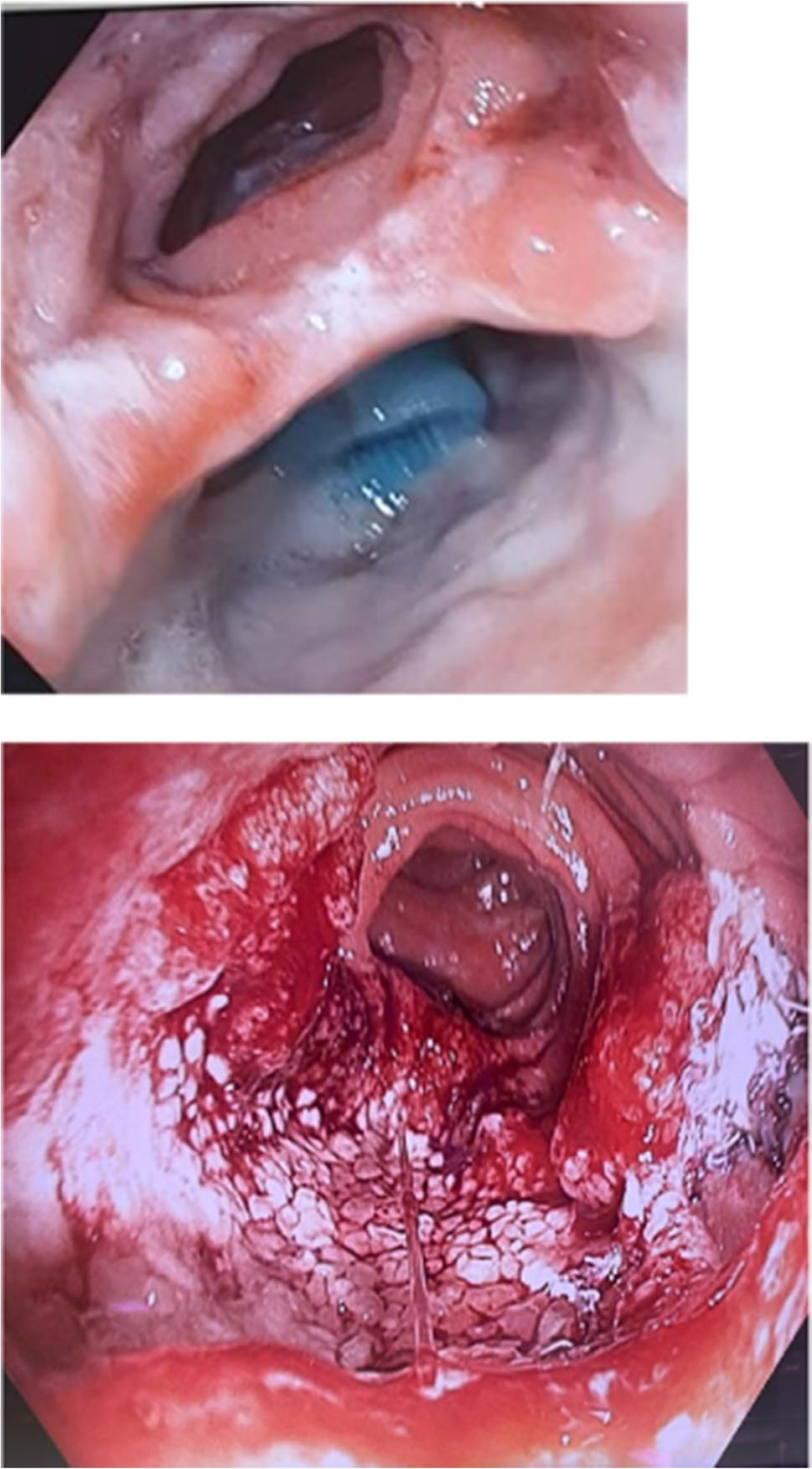
**a** Dehiscence at the gastro-jejunostomy. Next to the alimentary limb, the tips of the intraabdominal drains are visible in the area of the dehiscence. **b** Granulation tissue after 19 days of EVT

Figures 1a and b highlight an exemplary leak at the gastro-jejunal anastomosis from this small case series, before and after successful EVT for 19 days. Further information regarding therapeutic management and outcome is demonstrated in Table 3.

**Table 3:** Therapeutic procedure and outcome

	*n* (% or SD/median)
Therapeutic strategy	
Standalone EVT	3 (14.3%)
Combination of diagnostic laparoscopy + EVT	18 (85.7%)
Intermittent SEMS therapy	16 (76.2%)
Prior to EVT (outward MBS clinic)	6 (37.5%)
Prior to EVT (blowout fistula)	5 (31.25%)
After EVT	5 (31.25%)
Interval index surgery – first revision (days)	7.0 (0–38)
Interval diagnosis - EVT application (days)	27.5 (0–89)
Frequency EVT replacements (*n*)	6 (0–33)
EVT placement technique	
Overtube technique	16 (76.2%)
Pull-through technique	5 (23.8%)
Mean EVT dwell time (days)	25.7 (± 22.4)
EVT complications	
Dislocation	1 (4.7%)
Discontinuation due to patient’s incompliance	3 (14.3%)
Admission ICU	
Necessity for ICU (yes)	18 (85.7%)
Length of ICU stay (days)	26.4 (± 25.2)
Lenght of hospital stay (days)	77.3 (± 61.2)
Follow-up (median)	780.3 (99–2176)
Follow-up (complications/side effects)	
Dysphagia with pneumatic dilatation	3 (14.3%)
Persistant enterocutaneous fistula	1 (4.7%)
Pleural empyema	2 (9.5%)
Depression	8 (38.0%)
Mortality	
< 90 days after index surgery	0 (0%)
> 90 days after index surgery	4 (19.0%)
Success with EVT	20 (95.2%)

*EVT*, endoluminal vacuum therapy; *SEMS*, self-expanding metal stent; *ICU*, intensive care unit; range days: median (minimum–maximum)

## Discussion

This small single-center study highlights the efficacy of EVT in the management of SLL or AL after MBS with a success rate of 95.2%. Although postoperative leakage of the staple line [19] or anastomosis is a rare complication after MBS, management is still very challenging due to the extended risk of morbidity and mortality in this fragile group of patients [4]. Given the anatomical proximity to the pleura and mediastinum, a prompt and appropriate treatment is mandatory [20]. Considering the proven efficacy of EVT as an effective endoscopic approach for managing anastomotic leakage after both, colorectal and esophageal surgeries, its application in MBS appears to be a logical consequence in selected cases [21, 22]. This study highlights a long observational period of more than 7 years (01/2016–08/2023) with EVT treating SLL or AL successfully. This study showed that EVT represents an effective and safe therapeutic option in SLL or AL after MBS and an attractive add-on or alternative to revisional surgery or the use of SEMS therapy [17, 19, 23, 24].

### Patients’ Cohort

Distribution of gender and age in this study was similar to previously published literature regarding MBS [17]. Even though the mean age was relatively young, patients suffered from multiple obesity-associated comorbidities at the time of surgery, representing a fragile patient population. These patients run a higher risk of developing complications, which tend to be more complex to treat. The same is true for two patients that were suffering from severe hypoabsorption and liver dysfunction at the time of admission. The fact that half of the patients acquired a leak after a secondary bariatric procedure shows the complexity of these revisional procedures. Also, the need for specific bariatric centers with expertise and knowledge in the treatment of these patients can clearly be seen as 57% of patients underwent index surgery at an outward bariatric center and were transferred for further treatment. On a side note, it should be mentioned that all revisional surgeries and endoscopic interventions at the MUV are always performed by the same group of general surgeons, specialized in MBS, providing high quality with critical cases.

### Clinical Presentation

In a publication by Archid et al. analyzing SLL after sleeve gastrectomy, leakage occurred in 1.6%. Symptoms in this study occurred after a period of 22.8 (± 15.8) days and the length of hospital stay increased to 27.5 (± 20.4) days after MBS [19]. In the present case series, the onset of symptoms was after an average of 4.5 days post-index surgery, still during hospital stay in most cases, with a time to diagnosis of 6.0 days. However, in some of the patients transferred from an outward hospital, a delay of some days was observed when starting EVT. Also, some patients, especially after SG, came into the walk-in clinic with fever and severe abdominal pain several days after the index surgery. Due to the delayed diagnosis, local peritonitis or abscess often already existed, making sufficient therapy even more challenging. The radiological diagnosis of the leakage with slightly over 80% was comparable to the findings of Archid et al. [19] but shows that clinical presentation is as important as a radiological proof of leakage when starting therapy of the complication.

### Therapeutic Procedure

Since more than 50% of patients treated for SLL or AL at our institution initially underwent bariatric (revisional) surgery in other MBS centers, the time to implementing EVT was delayed in some cases. This group of patients underwent revisional surgery or SEMS therapy at the primary bariatric center and none of them were treated with EVT until they were referred to the MUV. Also, six patients received SEMS during revisional surgery at outward MBS centers, leading to the assumption that SEMS application might be easier or more established than EVT. However, a prompt start of EVT—for example, during index endoscopy—could be an ideal treatment strategy for one-stage diagnosis and treatment [19]. This could be combined either with diagnostic laparoscopy and drainage of the leakage or with CT-guided drainage of intra-abdominal abscess/fluid collections. In our cohort, standalone EVT was applied in only three patients, whereas in most cases, a combined approach of revisional surgery and EVT was preferred. An intermediate SEMS therapy was applied in changing the therapeutic modality (see Table 3).

EVT might be superior to SEMS therapy due to the negative pressure it creates, which makes drainage toward the luminal cavity possible. This establishes EVT as the preferred method in cases of late leaks, where the imperative is not to seal the leak but to actively promote secondary tissue healing [24]. In the future, a feasible and interesting therapeutic alternative for SLL might be VacStent GI™, a combination of SEMS and EVT [25].

When comparing the present study’s findings to a meta-analysis by Intriago et al., similar results were observed regarding EVT system exchanges (6.47 vs. 6) and EVT system duration time (25.7 days vs. 25.1 days). However, while EVT system dislodgement in the meta-analysis was observed in 12.5%, it was found in only one case in the current study [19].

Contrary to SEMS, where several complications such as stent migration and dislocation, damage of the digestive wall, stenosis, low tolerability, and high rates of dysphagia have been reported on [22], no severe complications occurred during EVT in this study. However, there are two main disadvantages to EVT. For one, EVT needs to be discontinued in some cases due to patient’s non-compliance regarding strict fasting requirements—as happened in three cases in the present study. Second, EVT implies a prolonged treatment duration due to multiple EVT replacements under general anesthesia.

### Outcome

During patient’s follow-up, three (*n* = 3/21; 14.3%) patients in this study developed dysphagia with the necessity for pneumatic dilatation. This is not unusual as intraluminal scar tissue can lead to a relative stenosis; however, endoscopic pneumatic dilatation was necessary in one case only.

Regarding the four patients who passed away within the follow-up period, their death was linked to different diseases including sepsis, kidney dysfunction and prolonged weaning, short bowel syndrome, but not to the leak itself in all but one case. Also, none of these was directly linked to EVT.

Therefore, these satisfying results indicate a therapeutic success of over 90% and support data of previously published case series with success rates between 80 and 100% [17, 19, 23, 24].

## Findings and Considerations

EVT was equally successful in acute leaks as well as in early leaks. No late or chronic leaks were observed regarding the classification by Rosenthal et al. [18]. Our findings, also supported by other published case series, indicate that EVT represents a secure and efficacious therapeutic option for SLL or AL, following MBS. It is a promising alternative to revisional surgery or the application of SEMS therapy [17, 19, 23, 24]. EVT seems to be more effective in situations where a wound cavity exists, than in chronic enterocutaneous fistulas. Considering EVT early or even preventive may be considered, with an emphasis on comprehensive training in its administration at outward surgical centers performing MBS.

EVT demands a certain degree of tolerance and patience, especially due to the long dwell time, multiple system exchanges, and long hospitalization. Also, even though fasting and nicotine abstinence were obligatory, a lack of compliance was observed in some patients. Therefore, a good doctor-patient relationship is beneficial for therapeutic outcome.

In order to detect leakage early, it is important to critically observe the clinical presentation of patients, even when diagnostic assessments fail to provide clear directives. The criteria for initiating re-laparoscopy in patients with suspected complications after MBS should be established with a low threshold [26]. To support patients’ mental health and boost compliance during their hospital stay and during follow-up at the outpatient clinic, providing psychological support early is important.

## Strengths and Limitations of This Study

An enormous strength of this study is that all patients were treated by the same surgical team, specialized in MBS, performing revisional surgery as well as endoscopy and EVT placement themselves. EVT represents a minimally invasive and safe therapeutic alternative to revisional surgery with reduced morbidity and promising therapeutic results. Changes in therapeutic strategy were discussed within the team and adapted to patient’s individual clinical state, need, and compliance. A limitation of this retrospective analysis is the rather small and heterogenous patient cohort with various comorbidities and different types of bariatric operations, resulting in challenging and diverse therapeutic management, as well as difficulties in data workup regarding patients’ and clinical characteristics. Especially if patients had index surgery at an outward bariatric center, data regarding leakage diagnosis and early postoperative management were partially incomplete. Furthermore, even though a life-long follow-up in the outpatient clinic is obligatory at defined time intervals, high lost-to-follow-up was observed. Not only due to the small size of this case series, but also due to the heterogenous therapeutic approaches, especially taken in outward MBS centers, it was not possible to define an optimal therapeutic algorithm for patients with leakage after MBS. Most importantly, since 16/21 patients were treated not only with EVT but also SEMS or re-operation during their therapeutic course, it remains unclear whether the therapeutic effect after sufficient wound granulation was achieved solely due to EVT. Nevertheless, all patients receiving EVT after MBS were reported in this case series and change in therapeutic strategy due to individual patients’ management and compliance reflects a normal therapeutic process.

## Conclusion

EVT proves to be a secure and minimally invasive therapeutic option, serving as a sufficient and effective addition to surgical intervention in cases of post-MBS leakage, with a clinical success rate of over 90% in this small case series. The collected information supports our tendency to establish EVT even more and earlier in our daily clinical practice when leakage after MBS occurs, avoiding reoperation in some patients and preventing further morbidity and mortality in critically ill patients. All surgeons in specialized MBS centers should implement EVT into their treatment armamentarium in complex cases.

## Abbreviations

AL: Anastomotic leakage
BMI: Body mass index (kg/m^2^)
CT: Computed tomography
EVT: Endoscopic vacuum therapy
GB: Gastric bypass
GI: Gastrointestinal
ICU: Intensive care unit
MBS: Metabolic/bariatric surgery
MUV: Medical University of Vienna
NaCl: Sodium chloride
RYGB: Roux-en-Y gastric bypass
SEMS: Self-expanding metal stent
SG: Sleeve gastrectomy
SLL: Staple-line leakage
